# Supplementary material for: Responses of marine trophic levels to the combined effects of ocean acidification and warming
Source: Nat Commun. 2024 Apr 22;15:3400. doi: 10.1038/s41467-024-47563-3 (PMC11035698; doi:10.1038/s41467-024-47563-3)
Supplement: Supplementary file 1 — Supplementary Information [file 41467_2024_47563_MOESM1_ESM.pdf]

# Supplementary Information

**Responses of Marine Trophic Levels to the Combined Effects of Ocean Acidification and Warming**

Nan Hu[1], Paul E. Bourdeau[2], Johan Hollander[3*]

[1]Department of Biology- Aquatic ecology, Lund University, Lund, Sweden

[2]Department of Biological Sciences, California State Polytechnic University, Humboldt, Arcata, California, USA

[3]World Maritime University, Ocean Sustainability, Governance & Management Unit, 211 18 Malmö, Sweden

[*]Correspondence: Johan Hollander, World Maritime University, Malmö, Sweden.

e-mail: johan.hollander@wmu.se

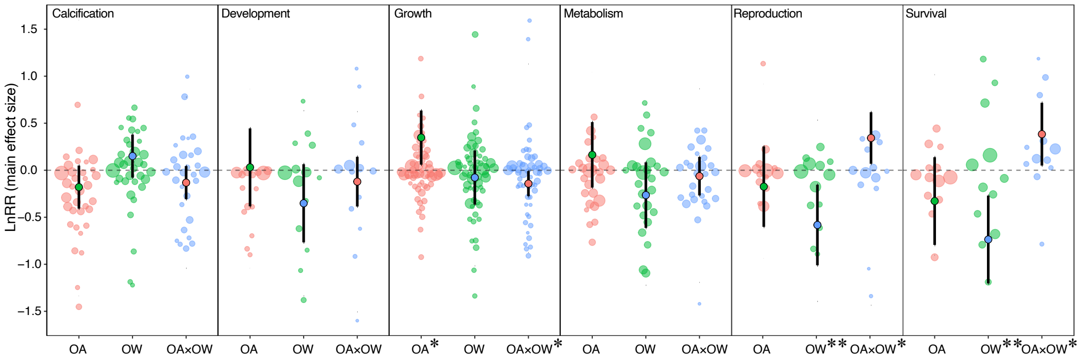


**Supplementary Fig 1.** Orchard plots showing the mean effect size, confidence interval (CIs, bold line) and individual effect sizes with precision (inverse variance) for ocean acidification (red), ocean warming (green), and their combined effects (blue) on different biological responses. Mean effect sizes and 95% confidence intervals were estimated from multi-level meta-analytic models (two-sided) using the stressor and biological responses as moderators. 95% confidence interval do not overlap with zero, indicating significant effect showing by asterisk ($0.01 < {}^{*}p < 0.05$; $0.001 < {}^{**}p < 0.01$; ${}^{***}p < 0.001$).

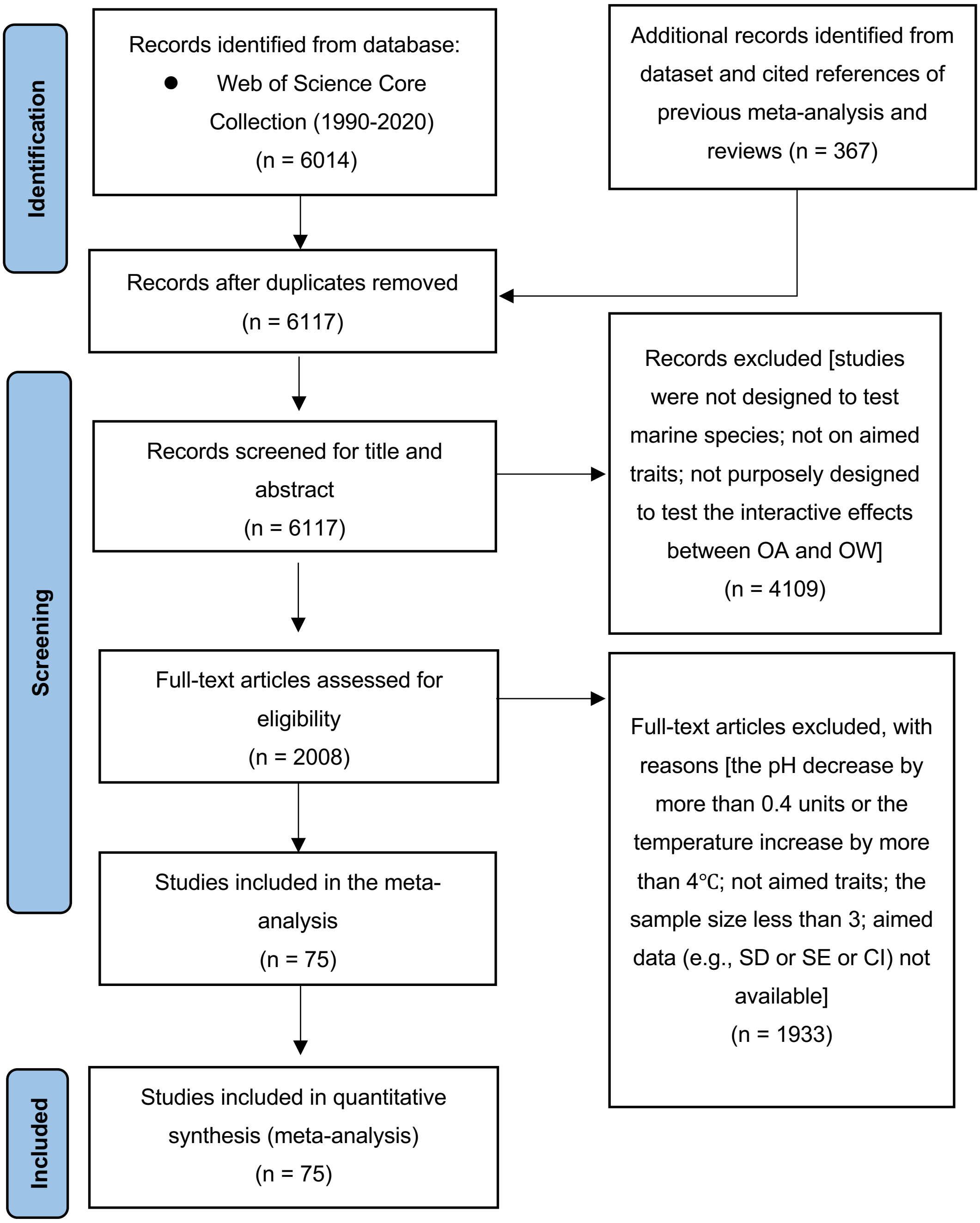


**Supplementary Fig 2. PRISMA flow diagram showing the process for locating publications included in this meta-analysis.**

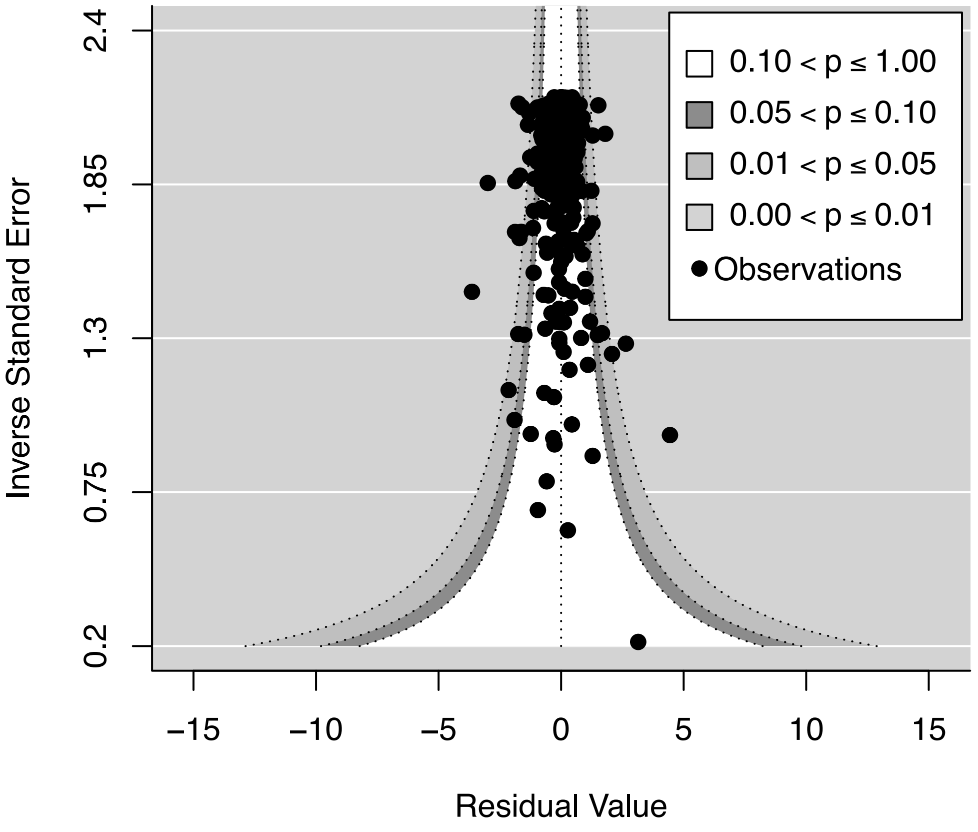


**Supplementary Fig 3. Assessment of publication bias.** Funnel plots show lnRR effect size against their inverse standard error for the dataset. The funnel plot was generated from the best model including the calcifier as the moderator (Supplementary Table 2), with the optimal random effects structure (Supplementary Table 1). Symmetrical points around the mean effect (vertical line) indicate no bias. There is little evidence of publication bias.

**Supplementary Table 1 Model selection for optimal random effects structure.** Random effects including: study ID (unique identifier per publication), species ID (unique identifier per species), individual effect size ID (to quantify residual heterogeneity), country (unique identifier per country) in which the experiment was performed (if this information was not included in the study, we used the country of the first author), and author group that studies shared the first author. Optimal random effects structure is denoted in bold. Models were fitted with maximum likelihood.

| Random effects | AIC |
| --- | --- |
| Study ID, Effect Size ID (residual) | **846.65** |
| Species, Study ID, Effect Size ID (residual) | 848.66 |
| Country, Study ID, Effect Size ID (residual) | 848.65 |
| Shared first author, Study ID, Effect Size ID (residual) | 848.59 |
| Country, Shared first author, Study ID, Effect Size ID (residual) | 850.56 |
| Species, Shared first author, Study ID, Effect Size ID (residual) | 850.69 |
| Species, Country, Study ID, Effect Size ID (residual) | 850.65 |
| Country, Species, Shared first author, Study ID, Effect Size ID (residual) | 852.57 |

**Supplementary Table 2 Model selection for evaluating publication bias.** Moderators included: Trophic Level – a factor with 4 levels referring to trophic levels; Stressor – a factor with 3 levels (OA, OW and OA×OW); Calcifier – a factor with 2 levels (calcifiers and non-calcifiers). The best model is denoted with bold. Models were fitted with maximum likelihood.

| Moderator | Random effects | AIC |
|---|---|---|
| Stressor | Study ID, Effect Size ID | 850.44 |
| Trophic Level | Study ID, Effect Size ID | 851.27 |
| Calcifier | Study ID, Effect Size ID | **849.51** |
| Calcifier + Trophic Level | Study ID, Effect Size ID | 853.16 |
| Calcifier + Stressor | Study ID, Effect Size ID | 852.23 |
| Stressor + Trophic Level | Study ID, Effect Size ID | 853.99 |
| Stressor + Trophic Level + Calcifier | Study ID, Effect Size ID | 855.88 |

**Supplementary Table 3. Heterogeneity tests of model fit.** Summary of heterogeneity tests (Q statistics) of multi-level meta-analytic models with different moderators. The total heterogeneity ($Q_T$) can be partitioned into heterogeneity of the structural model $Q_{Model}$ ($Q_M$) and unexplained heterogeneity $Q_{Error}$ ($Q_E$). $Q_M$ describes the amount of heterogeneity that can be explained by the model structure, and a significant $Q_E$ indicates there is still substantial variation unaccounted for by the model.

| | | Heterogeneity | | | |
|---|---|---|---|---|---|
| Dataset | Moderator | $Q_M$ | $P$ ($Q_M$) | $Q_E$ | $P(Q_E)$ |
| Overall | Stressor | 15.39 | 0.0015 | 14700.59 | <.0001 |
| | Stressor × Trophic Level | 22.28 | 0.0345 | 15152.85 | <.0001 |
| Calcifier | Stressor | 12.29 | 0.0065 | 9960.17 | <.0001 |
| | Stressor × Trophic Level | 13.47 | 0.3356 | 9669.07 | <.0001 |
| Non-calcifier | Stressor | 9.38 | 0.0246 | 4719.84 | <.0001 |
| | Stressor × Trophic Level | 22.76 | 0.0298 | 3737.78 | <.0001 |
| Tropical | Stressor | 7.12 | 0.0680 | 1251.56 | <.0001 |
| Sub-tropical | Stressor | 13.98 | 0.0029 | 7829.84 | <.0001 |
| Temperate | Stressor | 7.44 | 0.0590 | 5426.57 | <.0001 |

**Supplementary Table 4. Effects of ocean acidification, ocean warming, and their interaction on marine trophic levels.** Mean effect sizes and 95% confidence intervals were estimated from multi-level meta-analytic models (two-sided) that included trophic levels and stressors as the moderators, using main effect sizes. Percentages in parathesis denote the proportion of changes. *P*-values in bold denote a significant effect. *k* denotes the number of observations for each trophic level.

| Trophic Level | Stressor | Effect size | Lower CI | Upper CI | *k* | *p* |
|---|---|---|---|---|---|---|
| Pooled | OA | -0.11 (-10.4%) | -0.185 (-16.9%) | -0.034 (-3.4%) | 162 | **0.0043** |
| Pooled | OW | -0.106 (-10%) | -0.182 (-16.6%) | -0.03 (-2.9%) | 162 | **0.0065** |
| Pooled | OA×OW | -0.053 (-5.1%) | -0.138 (-12.9%) | 0.033 (3.3%) | 162 | 0.2261 |
| Primary Producer | OA | 0.195 (21.5%) | -0.051 (-5%) | 0.441 (55.4%) | 58 | 0.1205 |
| Primary Producer | OW | 0.007 (0.7%) | -0.239 (-21.3%) | 0.254 (28.9%) | 58 | 0.9533 |
| Primary Producer | OA×OW | -0.063 (-6.1%) | -0.256 (-22.6%) | 0.13 (13.8%) | 58 | 0.5202 |
| Herbivore | OA | -0.171 (-15.8%) | -0.282 (-24.6%) | -0.061 (-5.9%) | 75 | **0.0024** |
| Herbivore | OW | -0.106 (-10.1%) | -0.218 (-19.6%) | 0.006 (0.6%) | 75 | 0.0636 |
| Herbivore | OA×OW | -0.041 (-4%) | -0.167 (-15.4%) | 0.085 (8.8%) | 75 | 0.5208 |
| Meso-predator | OA | -0.003 (-0.3%) | -0.341 (-28.9%) | 0.336 (39.9%) | 20 | 0.9875 |
| Meso-predator | OW | -0.013 (-1.3%) | -0.352 (-29.7%) | 0.326 (38.5%) | 20 | 0.9396 |
| Meso-predator | OA×OW | 0.059 (6%) | -0.199 (-18%) | 0.316 (37.2%) | 20 | 0.6549 |
| Top-predator | OA | 0.115 (12.2%) | -0.392 (-32.5%) | 0.622 (86.3%) | 9 | 0.6570 |
| Top-predator | OW | 0.225 (25.2%) | -0.283 (-24.7%) | 0.733 (108%) | 9 | 0.3858 |
| Top-predator | OA×OW | 0.047 (4.8%) | -0.362 (-30.4%) | 0.456 (57.8%) | 9 | 0.8227 |
| Predator | OA | 0.032 (3.3%) | -0.267 (-23.4%) | 0.331 (39.3%) | 29 | 0.8327 |
| Predator | OW | 0.057 (5.8%) | -0.243 (-21.5%) | 0.356 (42.8%) | 29 | 0.7099 |
| Predator | OA×OW | 0.052 (5.3%) | -0.179 (-16.4%) | 0.283 (32.6%) | 29 | 0.6617 |

**Supplementary Table 5. Effects of ocean acidification, ocean warming, and their interaction on marine trophic levels of calcifiers.** Mean effect sizes and 95% confidence intervals were estimated from multi-level meta-analytic models (two-sided) that included the trophic level and the stressor as the moderators using main effect sizes. Percentages in parathesis denote the proportion of changes. *P*-values in bold denote a significant effect. *k* denotes the number of observations for each trophic level.

| Trophic Level | Stressor | Effect size | Lower CI | Upper CI | *k* | *p* |
|---|---|---|---|---|---|---|
| Pooled | OA | -0.165 (-15.2%) | -0.266 (-23.4%) | -0.064 (-6.2%) | 113 | **0.0014** |
| Pooled | OW | -0.073 (-7%) | -0.175 (-16%) | 0.029 (2.9%) | 113 | 0.1594 |
| Pooled | OA×OW | -0.056 (-5.4%) | -0.169 (-15.5%) | 0.057 (5.9%) | 113 | 0.3320 |
| Primary Producer | OA | 0.063 (6.5%) | -0.281 (-24.5%) | 0.407 (50.2%) | 33 | 0.7202 |
| Primary Producer | OW | 0.052 (5.4%) | -0.293 (-25.4%) | 0.397 (48.7%) | 33 | 0.7665 |
| Primary Producer | OA×OW | -0.038 (-3.7%) | -0.306 (-26.3%) | 0.23 (25.8%) | 33 | 0.7805 |
| Herbivore | OA | -0.176 (-16.1%) | -0.314 (-27%) | -0.038 (-3.7%) | 62 | **0.0125** |
| Herbivore | OW | -0.095 (-9.1%) | -0.235 (-20.9%) | 0.044 (4.5%) | 62 | 0.1793 |
| Herbivore | OA×OW | -0.043 (-4.2%) | -0.197 (-17.9%) | 0.111 (11.8%) | 62 | 0.5851 |
| Meso-predator | OA | 0.036 (3.7%) | -0.444 (-35.9%) | 0.516 (67.6%) | 12 | 0.8830 |
| Meso-predator | OW | 0.06 (6.2%) | -0.421 (-34.3%) | 0.54 (71.6%) | 12 | 0.8073 |
| Meso-predator | OA×OW | -0.025 (-2.5%) | -0.384 (-31.9%) | 0.334 (39.6%) | 12 | 0.8910 |
| Top-predator | OA | 0.043 (4.4%) | -0.651 (-47.8%) | 0.736 (108.8%) | 6 | 0.9040 |
| Top-predator | OW | 0.275 (31.6%) | -0.42 (-34.3%) | 0.969 (163.6%) | 6 | 0.4383 |
| Top-predator | OA×OW | 0.019 (2%) | -0.531 (-41.2%) | 0.57 (76.9%) | 6 | 0.9451 |
| Predator | OA | 0.04 (4.1%) | -0.373 (-31.1%) | 0.454 (57.4%) | 18 | 0.8487 |
| Predator | OW | 0.129 (13.8%) | -0.285 (-24.8%) | 0.543 (72.1%) | 18 | 0.5406 |
| Predator | OA×OW | -0.017 (-1.7%) | -0.331 (-28.2%) | 0.298 (34.7%) | 18 | 0.9161 |

**Supplementary Table 6. Effects of ocean acidification, ocean warming, and their interaction on marine trophic levels of non-calcifiers.** Mean effect sizes and 95% confidence intervals were estimated from multi-level meta-analytic models (two-sided) that included the trophic level and the stressor as the moderators using main effect sizes. Percentages in parathesis denote the proportion of changes. *P*-values in bold denote a significant effect. *k* denotes the number of observations for each trophic level.

| Trophic Level | Stressor | Effect size | Lower CI | Upper CI | *k* | *p* |
|---|---|---|---|---|---|---|
| Pooled | OA | 0.028 (2.8%) | -0.082 (-7.8%) | 0.137 (14.7%) | 49 | 0.6181 |
| Pooled | OW | -0.161 (-14.9%) | -0.271 (-23.7%) | -0.051 (-5%) | 49 | **0.0041** |
| Pooled | OA×OW | -0.037 (-3.6%) | -0.163 (-15.1%) | 0.09 (9.4%) | 49 | 0.5716 |
| Primary Producer | OA | 0.365 (44%) | 0.013 (1.3%) | 0.716 (104.7%) | 25 | **0.0419** |
| Primary Producer | OW | 0.003 (0.3%) | -0.349 (-29.4%) | 0.354 (42.5%) | 25 | 0.9879 |
| Primary Producer | OA×OW | -0.098 (-9.3%) | -0.405 (-33.3%) | 0.209 (23.2%) | 25 | 0.5309 |
| Herbivore | OA | -0.144 (-13.4%) | -0.354 (-29.8%) | 0.066 (6.8%) | 13 | 0.1781 |
| Herbivore | OW | -0.151 (-14%) | -0.363 (-30.5%) | 0.062 (6.4%) | 13 | 0.1646 |
| Herbivore | OA×OW | -0.028 (-2.8%) | -0.281 (-24.5%) | 0.225 (25.3%) | 13 | 0.8286 |
| Meso-predator | OA | -0.067 (-6.4%) | -0.51 (-40%) | 0.377 (45.8%) | 8 | 0.7686 |
| Meso-predator | OW | -0.078 (-7.5%) | -0.521 (-40.6%) | 0.366 (44.1%) | 8 | 0.7310 |
| Meso-predator | OA×OW | 0.163 (17.7%) | -0.215 (-19.3%) | 0.54 (71.6%) | 8 | 0.3984 |
| Top-predator | OA | 0.223 (25%) | -0.468 (-37.4%) | 0.914 (149.5%) | 3 | 0.5273 |
| Top-predator | OW | 0.206 (22.8%) | -0.485 (-38.4%) | 0.896 (145%) | 3 | 0.5597 |
| Top-predator | OA×OW | 0.154 (16.7%) | -0.463 (-37.1%) | 0.772 (116.4%) | 3 | 0.6239 |
| Predator | OA | 0.006 (0.6%) | -0.407 (-33.4%) | 0.419 (52%) | 11 | 0.9778 |
| Predator | OW | -0.006 (-0.6%) | -0.419 (-34.2%) | 0.406 (50.1%) | 11 | 0.9755 |
| Predator | OA×OW | 0.157 (17%) | -0.201 (-18.2%) | 0.515 (67.4%) | 11 | 0.3891 |

**Supplementary Table 7. Relationship between the absolute latitude and main effect sizes.** Results were generated from multi-level meta-regression using absolute latitude as the moderator. Parameters that were significantly different from zero denoted as bold. SE: standard error.

| Trophic Level | Stressor | *df* | Intercept | *SE* | *p* | Slope | *SE* | *p* |
|---|---|---|---|---|---|---|---|---|
| Primary Producer | OA | 57 | -0.0668 | 0.1613 | 0.6790 | 0.0011 | 0.0041 | 0.7846 |
| Primary Producer | OW | 57 | -0.6457 | 0.1960 | **0.0010** | 0.0138 | 0.0050 | **0.0059** |
| Primary Producer | OA×OW | 57 | -0.1393 | 0.2118 | 0.5106 | 0.0010 | 0.0054 | 0.8589 |
| Herbivore | OA | 74 | -0.3593 | 0.1391 | **0.0098** | 0.0048 | 0.0033 | 0.1421 |
| Herbivore | OW | 74 | -0.1497 | 0.2139 | 0.4840 | 0.0009 | 0.0050 | 0.8528 |
| Herbivore | OA×OW | 74 | 0.5153 | 0.4147 | 0.2139 | -0.0126 | 0.0097 | 0.1934 |
| Meso-predator | OA | 19 | -0.0828 | 0.2338 | 0.7233 | -0.0007 | 0.0060 | 0.9066 |
| Meso-predator | OW | 19 | -0.1182 | 0.2131 | 0.5792 | 0.0012 | 0.0055 | 0.8232 |
| Meso-predator | OA×OW | 19 | 0.2223 | 0.2451 | 0.3643 | -0.0054 | 0.0063 | 0.3961 |
| Top-predator | OA | 8 | 0.6789 | 0.3093 | **0.0282** | -0.0142 | 0.0066 | **0.0313** |
| Top-predator | OW | 8 | 0.0599 | 2.0360 | 0.9765 | 0.0025 | 0.0441 | 0.9540 |
| Top-predator | OA×OW | 8 | 0.0911 | 0.5452 | 0.8673 | -0.0013 | 0.0112 | 0.9082 |
| Predator | OA | 28 | -0.0478 | 0.2070 | 0.8176 | -0.0007 | 0.0051 | 0.8866 |
| Predator | OW | 28 | -0.1436 | 0.3169 | 0.6503 | 0.0035 | 0.0078 | 0.6555 |
| Predator | OA×OW | 28 | 0.1959 | 0.1949 | 0.3149 | -0.0042 | 0.0048 | 0.3788 |

**Supplementary Table 8. Effects of ocean acidification, ocean warming, and their interaction on marine species across different climate regions.** Mean effect sizes and 95% confidence intervals were estimated from multi-level meta-analytic models (two-sided) that included the stressor as the moderator using main effect sizes. Percentages in parathesis denote the proportion of changes. *P*-values in bold denote a significant effect. *k* denotes the number of observations for each trophic level.

| Region | Stressor | Effect size | Lower CI | Upper CI | *k* | *p* |
|---|---|---|---|---|---|---|
| Tropical | OA | -0.038 (-3.7%) | -0.516 (-40.3%) | 0.441 (55.4%) | 17 | 0.8767 |
| Tropical | OW | -0.119 (-11.2%) | -0.597 (-45.0%) | -0.359 (43.2%) | 17 | 0.3592 |
| Tropical | OA×OW | 0.560 (75.1%) | 0.062 (6.2%) | 1.059 (188.3%) | 17 | **0.0277** |
| Sub-tropical | OA | -0.164 (-15.1%) | -0.288(-25.0%) | -0.039 (-3.8%) | 67 | **0.0100** |
| Sub-tropical | OW | -0.176 (-16.1%) | -0.302(-26.1%) | -0.050 (-4.8%) | 67 | **0.0063** |
| Sub-tropical | OA×OW | -0.081 (-7.8%) | -0.223 (-20.0%) | 0.061 (6.3%) | 67 | 0.2642 |
| Temperate | OA | -0.057 (-5.6%) | -0.146 (-13.6%) | 0.031 (3.2%) | 78 | 0.2042 |
| Temperate | OW | -0.029 (-2.9%) | -0.118(-11.1%) | 0.059 (6.1%) | 78 | 0.5194 |
| Temperate | OA×OW | -0.129 (-12.1%) | -0.229 (-20.5%) | -0.028 (-2.8%) | 78 | **0.0121** |

**Supplementary Table 9. Sensitivity analysis according to Figure 2 and Supplementary Table 4.** Results were generated from models using the variance-covariance matrices with a correlation of 0.9 among correlated effect size sample variances. See more details in the legend of Supplementary Table 4.

| Trophic Level | Stressor | Effect size | Lower CI | Upper CI | *k* | *p* |
|---|---|---|---|---|---|---|
| Pooled | OA | -0.113 (-10.7%) | -0.195 (-17.7%) | -0.031 (-3%) | 162 | **0.0071** |
| Pooled | OW | -0.1 (-9.5%) | -0.183 (-16.7%) | -0.018 (-1.7%) | 162 | **0.0175** |
| Pooled | OA×OW | -0.049 (-4.8%) | -0.142 (-13.3%) | 0.044 (4.5%) | 162 | 0.3036 |
| Primary Producer | OA | 0.203 (22.4%) | -0.058 (-5.7%) | 0.463 (59%) | 58 | 0.1283 |
| Primary Producer | OW | 0.009 (0.9%) | -0.252 (-22.3%) | 0.27 (31%) | 58 | 0.9453 |
| Primary Producer | OA×OW | -0.07 (-6.7%) | -0.28 (-24.4%) | 0.141 (15.1%) | 58 | 0.5160 |
| Herbivore | OA | -0.177 (-16.2%) | -0.298 (-25.7%) | -0.056 (-5.4%) | 75 | **0.0041** |
| Herbivore | OW | -0.099 (-9.5%) | -0.221 (-19.9%) | 0.022 (2.3%) | 75 | 0.1094 |
| Herbivore | OA×OW | -0.032 (-3.1%) | -0.17 (-15.6%) | 0.106 (11.2%) | 75 | 0.6515 |
| Meso-predator | OA | 0.013 (1.3%) | -0.354 (-29.8%) | 0.379 (46.1%) | 20 | 0.9461 |
| Meso-predator | OW | -0.01 (-1%) | -0.376 (-31.3%) | 0.356 (42.8%) | 20 | 0.9582 |
| Meso-predator | OA×OW | 0.051 (5.2%) | -0.232 (-20.7%) | 0.334 (39.7%) | 20 | 0.7237 |
| Top-predator | OA | 0.149 (16.1%) | -0.379 (-31.6%) | 0.678 (96.9%) | 9 | 0.5805 |
| Top-predator | OW | 0.269 (30.9%) | -0.259 (-22.8%) | 0.798 (122%) | 9 | 0.3174 |
| Top-predator | OA×OW | 0.018 (1.8%) | -0.426 (-34.7%) | 0.462 (58.7%) | 9 | 0.9375 |
| Predator | OA | 0.057 (5.8%) | -0.264 (-23.2%) | 0.377 (45.8%) | 29 | 0.7284 |
| Predator | OW | 0.078 (8.1%) | -0.242 (-21.5%) | 0.398 (48.9%) | 29 | 0.6331 |
| Predator | OA×OW | 0.035 (3.6%) | -0.218 (-19.6%) | 0.289 (33.4%) | 29 | 0.7852 |

**Supplementary Table 10. Sensitivity analysis on calcifiers according to Figure 3a and Supplementary Table 5.** Results were generated from models using the variance-covariance matrices, with a correlation of 0.9 among correlated effect size sample variances. See more details in the legend of Supplementary Table 5.

| Trophic Level | Stressor | Effect size | Lower CI | Upper CI | *k* | *p* |
|---|---|---|---|---|---|---|
| Pooled | OA | -0.113 (-10.7%) | -0.195 (-17.7%) | -0.031 (-3%) | 162 | **0.0071** |
| Pooled | OW | -0.1 (-9.5%) | -0.183 (-16.7%) | -0.018 (-1.7%) | 162 | **0.0175** |
| Pooled | OA×OW | -0.049 (-4.8%) | -0.142 (-13.3%) | 0.044 (4.5%) | 162 | 0.3036 |
| Primary Producer | OA | 0.203 (22.4%) | -0.058 (-5.7%) | 0.463 (59%) | 58 | 0.1283 |
| Primary Producer | OW | 0.009 (0.9%) | -0.252 (-22.3%) | 0.27 (31%) | 58 | 0.9453 |
| Primary Producer | OA×OW | -0.07 (-6.7%) | -0.28 (-24.4%) | 0.141 (15.1%) | 58 | 0.516 |
| Herbivore | OA | -0.177 (-16.2%) | -0.298 (-25.7%) | -0.056 (-5.4%) | 75 | **0.0041** |
| Herbivore | OW | -0.099 (-9.5%) | -0.221 (-19.9%) | 0.022 (2.3%) | 75 | 0.1094 |
| Herbivore | OA×OW | -0.032 (-3.1%) | -0.17 (-15.6%) | 0.106 (11.2%) | 75 | 0.6515 |
| Meso-predator | OA | 0.013 (1.3%) | -0.354 (-29.8%) | 0.379 (46.1%) | 20 | 0.9461 |
| Meso-predator | OW | -0.01 (-1%) | -0.376 (-31.3%) | 0.356 (42.8%) | 20 | 0.9582 |
| Meso-predator | OA×OW | 0.051 (5.2%) | -0.232 (-20.7%) | 0.334 (39.7%) | 20 | 0.7237 |
| Top-predator | OA | 0.149 (16.1%) | -0.379 (-31.6%) | 0.678 (96.9%) | 9 | 0.5805 |
| Top-predator | OW | 0.269 (30.9%) | -0.259 (-22.8%) | 0.798 (122%) | 9 | 0.3174 |
| Top-predator | OA×OW | 0.018 (1.8%) | -0.426 (-34.7%) | 0.462 (58.7%) | 9 | 0.9375 |
| Predator | OA | 0.057 (5.8%) | -0.264 (-23.2%) | 0.377 (45.8%) | 29 | 0.7284 |
| Predator | OW | 0.078 (8.1%) | -0.242 (-21.5%) | 0.398 (48.9%) | 29 | 0.6331 |
| Predator | OA×OW | 0.035 (3.6%) | -0.218 (-19.6%) | 0.289 (33.4%) | 29 | 0.7852 |

**Supplementary Table 11. Sensitivity analysis on non-calcifiers according to Figure 3b and Supplementary Table 6.** Results were generated from models using the variance-covariance matrices with a correlation of 0.9 among correlated effect size sample variances. See more details in the legend of Supplementary Table 6.

| Trophic Level | Stressor | Effect size | Lower CI | Upper CI | *k* | *p* |
|---|---|---|---|---|---|---|
| Pooled | OA | 0.028 (2.9%) | -0.084 (-8%) | 0.14 (15.1%) | 49 | 0.6211 |
| Pooled | OW | -0.173 (-15.9%) | -0.286 (-24.8%) | -0.06 (-5.9%) | 49 | **0.0026** |
| Pooled | OA×OW | -0.032 (-3.2%) | -0.164 (-15.1%) | 0.1 (10.5%) | 49 | 0.6333 |
| Primary Producer | OA | 0.389 (47.6%) | 0.027 (2.7%) | 0.752 (112%) | 25 | **0.0354** |
| Primary Producer | OW | 0.025 (2.5%) | -0.336 (-28.5%) | 0.386 (47.1%) | 25 | 0.8921 |
| Primary Producer | OA×OW | -0.131 (-12.3%) | -0.455 (-36.6%) | 0.193 (21.3%) | 25 | 0.4281 |
| Herbivore | OA | -0.143 (-13.3%) | -0.358 (-30.1%) | 0.073 (7.6%) | 13 | 0.1951 |
| Herbivore | OW | -0.153 (-14.2%) | -0.372 (-31%) | 0.066 (6.8%) | 13 | 0.1711 |
| Herbivore | OA×OW | 0.002 (0.2%) | -0.269 (-23.6%) | 0.272 (31.3%) | 13 | 0.9911 |
| Meso-predator | OA | -0.04 (-3.9%) | -0.505 (-39.7%) | 0.425 (53%) | 8 | 0.8658 |
| Meso-predator | OW | -0.049 (-4.8%) | -0.513 (-40.1%) | 0.415 (51.4%) | 8 | 0.8349 |
| Meso-predator | OA×OW | 0.137 (14.7%) | -0.264 (-23.2%) | 0.538 (71.3%) | 8 | 0.5035 |
| Top-predator | OA | 0.209 (23.3%) | -0.478 (-38%) | 0.896 (145%) | 3 | 0.5506 |
| Top-predator | OW | 0.117 (12.4%) | -0.57 (-43.5%) | 0.804 (123.5%) | 3 | 0.7387 |
| Top-predator | OA×OW | 0.208 (23.1%) | -0.442 (-35.7%) | 0.858 (135.9%) | 3 | 0.5305 |
| Predator | OA | 0.034 (3.5%) | -0.394 (-32.6%) | 0.462 (58.8%) | 11 | 0.8753 |
| Predator | OW | 0.003 (0.3%) | -0.423 (-34.5%) | 0.43 (53.8%) | 11 | 0.9876 |
| Predator | OA×OW | 0.139 (14.9%) | -0.24 (-21.4%) | 0.519 (68%) | 11 | 0.4722 |

**Supplementary Table 12. Sensitivity analysis according to Figure 4 and Supplementary Table 7.** Results were generated from models using the variance-covariance matrices with a correlation of 0.9 among correlated effect size sample variances. See more details in the legend of Supplementary Table 7.

| Trophic Level | Stressor | *df* | Intercept | *SE* | *p* | Slope | *SE* | *p* |
|---|---|---|---|---|---|---|---|---|
| Primary Producer | OA | 57 | -0.0654 | 0.1636 | 0.6891 | 0.0011 | 0.0041 | 0.7974 |
| Primary Producer | OW | 57 | -0.6450 | 0.1989 | **0.0012** | 0.0138 | 0.0051 | **0.0070** |
| Primary Producer | OA×OW | 57 | -0.1334 | 0.2235 | 0.5507 | 0.0006 | 0.0057 | 0.9216 |
| Herbivore | OA | 74 | -0.3578 | 0.1438 | 0.0128 | 0.0048 | 0.0034 | 0.1561 |
| Herbivore | OW | 74 | -0.1497 | 0.2114 | 0.4789 | 0.0009 | 0.0050 | 0.8574 |
| Herbivore | OA×OW | 74 | 0.4534 | 0.3957 | 0.2519 | -0.0116 | 0.0092 | 0.2096 |
| Meso-predator | OA | 19 | -0.0769 | 0.2331 | 0.7415 | -0.0008 | 0.0060 | 0.8873 |
| Meso-predator | OW | 19 | -0.1168 | 0.2116 | 0.5809 | 0.0013 | 0.0055 | 0.8148 |
| Meso-predator | OA×OW | 19 | 0.2204 | 0.2331 | 0.3443 | -0.0050 | 0.0060 | 0.4020 |
| Top-predator | OA | 8 | 0.8910 | 0.7561 | 0.2386 | -0.0196 | 0.0164 | 0.2336 |
| Top-predator | OW | 8 | 0.0567 | 2.0714 | 0.9782 | 0.0026 | 0.0449 | 0.9544 |
| Top-predator | OA×OW | 8 | -0.0311 | 0.5705 | 0.9565 | 0.0021 | 0.0123 | 0.8617 |
| Predator | OA | 28 | -0.0335 | 0.2096 | 0.8729 | -0.0012 | 0.0051 | 0.8173 |
| Predator | OW | 28 | -0.1405 | 0.3270 | 0.6673 | 0.0033 | 0.0080 | 0.6787 |
| Predator | OA×OW | 28 | 0.1905 | 0.1844 | 0.3016 | -0.0038 | 0.0045 | 0.4078 |

**Supplementary Table 13. Sensitivity analysis according to Figure 5 and Supplementary Table 8.** Results were generated from models using the variance-covariance matrices with a correlation of 0.9 among correlated effect size sample variances. See more details in the legend of Supplementary Table 8.

| Region | Stressor | Effect size | Lower CI | Upper CI | *k* | *p* |
|---|---|---|---|---|---|---|
| Tropical | OA | -0.103 (-9.8%) | -0.525 (-40.9%) | 0.319 (37.5%) | 17 | 0.6313 |
| Tropical | OW | -0.177 (-16.2%) | -0.599 (-45.1%) | 0.245 (37.8%) | 17 | 0.4110 |
| Tropical | OA×OW | 0.527 (69.3%) | 0.076 (7.9%) | 0.978 (165.8%) | 17 | **0.0220** |
| Sub-tropical | OA | -0.168 (-15.5%) | -0.305(-26.3%) | -0.032 (-3.2%) | 67 | **0.0154** |
| Sub-tropical | OW | -0.167 (-15.4%) | -0.306(-26.5%) | -0.029 (-2.9%) | 67 | **0.0178** |
| Sub-tropical | OA×OW | -0.084 (-8.0%) | -0.240 (-21.4%) | 0.072 (7.5%) | 67 | 0.2929 |
| Temperate | OA | -0.056 (-5.4%) | -0.151(-14.1%) | 0.039 (4.0%) | 78 | 0.2476 |
| Temperate | OW | -0.022 (-2.2%) | -0.117(-11.0%) | 0.073 (7.6%) | 78 | 0.6529 |
| Temperate | OA×OW | -0.129 (-12.1%) | -0.238 (-21.2%) | -0.021 (-2.0%) | 78 | **0.0199** |
