# Supplementary material for: Responses of marine trophic levels to the combined effects of ocean acidification and warming
Source: Nat Commun. 2024 Apr 22;15:3400. doi: 10.1038/s41467-024-47563-3 (PMC11035698; doi:10.1038/s41467-024-47563-3)
Supplement: Supplementary file 3 — Reporting Summary [file 41467_2024_47563_MOESM3_ESM.pdf]

nature portfolio

Corresponding author(s): Johan Hollander
Last updated by author(s): 20/03/24

Reporting Summary

Nature Portfolio wishes to improve the reproducibility of the work that we publish. This form provides structure for consistency and transparency in reporting. For further information on Nature Portfolio policies, see our Editorial Policies and the Editorial Policy Checklist.

Statistics

For all statistical analyses, confirm that the following items are present in the figure legend, table legend, main text, or Methods section.

n/a	Confirmed	
☐	☒	The exact sample size (*n*) for each experimental group/condition, given as a discrete number and unit of measurement
☐	☒	A statement on whether measurements were taken from distinct samples or whether the same sample was measured repeatedly
☐	☒	The statistical test(s) used AND whether they are one- or two-sided<br>*Only common tests should be described solely by name; describe more complex techniques in the Methods section.*
☐	☒	A description of all covariates tested
☐	☒	A description of any assumptions or corrections, such as tests of normality and adjustment for multiple comparisons
☐	☒	A full description of the statistical parameters including central tendency (e.g. means) or other basic estimates (e.g. regression coefficient) AND variation (e.g. standard deviation) or associated estimates of uncertainty (e.g. confidence intervals)
☐	☒	For null hypothesis testing, the test statistic (e.g. *F*, *t*, *r*) with confidence intervals, effect sizes, degrees of freedom and *P* value noted<br>*Give P values as exact values whenever suitable.*
☒	☐	For Bayesian analysis, information on the choice of priors and Markov chain Monte Carlo settings
☐	☒	For hierarchical and complex designs, identification of the appropriate level for tests and full reporting of outcomes
☐	☒	Estimates of effect sizes (e.g. Cohen's *d*, Pearson's *r*), indicating how they were calculated

Our web collection on statistics for biologists contains articles on many of the points above.

Software and code

Policy information about availability of computer code

Data collection	Literature survey was conducted in ISI Web of Science using the databases of "Web of Science Core Collection". We also cross checked the datasets and references of the previous meta-analyses and reviews for potential literature. Data were extracted from tables directly and figures using WebPlotDigitizer (v. 4.4) or GraphClick (v. 3.0). Data was structured and coded using Microsoft Excel spreadsheet.
Data analysis	All analyses were conducted using the package metafor (v. 4.3.0) in R software (v. 4.2.2). The R code used in this study have been deposited on Zenodo digital repository at https://doi.org/10.5281/zenodo.10198752.

For manuscripts utilizing custom algorithms or software that are central to the research but not yet described in published literature, software must be made available to editors and reviewers. We strongly encourage code deposition in a community repository (e.g. GitHub). See the Nature Portfolio guidelines for submitting code & software for further information.

Data

Policy information about availability of data

All manuscripts must include a data availability statement. This statement should provide the following information, where applicable:

- Accession codes, unique identifiers, or web links for publicly available datasets
- A description of any restrictions on data availability
- For clinical datasets or third party data, please ensure that the statement adheres to our policy

All data used in this study have been deposited on Zenodo digital repository at https://doi.org/10.5281/zenodo.10198734.

Research involving human participants, their data, or biological material

Policy information about studies with human participants or human data. See also policy information about sex, gender (identity/presentation), and sexual orientation and race, ethnicity and racism.

Reporting on sex and gender	Not applicable.
Reporting on race, ethnicity, or other socially relevant groupings	Not applicable.
Population characteristics	Not applicable.
Recruitment	Not applicable.
Ethics oversight	Not applicable.

Note that full information on the approval of the study protocol must also be provided in the manuscript.

Field-specific reporting

Please select the one below that is the best fit for your research. If you are not sure, read the appropriate sections before making your selection.

☐ Life sciences ☐ Behavioural & social sciences ☒ Ecological, evolutionary & environmental sciences

For a reference copy of the document with all sections, see nature.com/documents/nr-reporting-summary-flat.pdf

Ecological, evolutionary & environmental sciences study design

All studies must disclose on these points even when the disclosure is negative.

Study description	We conducted a meta-analysis to quantify the responses of marine trophic levels to climatic stressors. We used hierarchical multi-level meta-analysis models to examine whether marine species from different trophic levels demonstrate differential responses to the single and combine stress of ocean acidification and ocean warming. We also tested if the distribution of interaction types (i.e., additive, synergistic, and antagonistic) differs across trophic levels. We additionally assessed how stressors, individually and in combination, influence marine species along a latitudinal gradient and among climate regions. We used a unique large data set including 486 observations from 162 fully factorial experiments.
Research sample	We followed the PRISMA protocol for study selection and inclusion in the systematic review and meta-analysis. Quantitative analyses were performed for 486 effect sizes from 75 studies.
Sampling strategy	The literature was searched through ISI Web of Science Core Collection. In addition to our literature survey, we also performed a cross-reference check of our database with the literature used and cited in the previous meta-analysis and reviews focusing on ocean acidification and/or warming.
Data collection	Nan Hu conducted the literature search, screened all literature, extracted all the effect sizes, and coded the information from the studies.
Timing and spatial scale	The literature search was conducted in 21st of April 2021. Following the peer-review process, we updated our search string. The included literature were published between 2003 and 2020. Year and geographical (Latitude and longitude) information are reported in the dataset deposited on Zenodo digital repository at https://doi.org/10.5281/zenodo.10198734.
Data exclusions	In the literature, we focused on the following organismal response variables: calcification, development, growth, metabolism, reproduction, and survival (mortality data were transformed to survival with [1 - mortality]). For multiple experimental treatments (where more than one treatment group was compared to the control group), we included the highest testing value that was within the range of the RCP 8.5 scenario, for example, if an experiment simultaneously included 2 degree- and 4 degree-increase treatments, the 4 degree-increase treatment was selected. When several measurements were taken for one response variable (i.e., body mass and length for growth), we only used the most inclusive one13,48. For studies with time series experiments, we only included the response reported at the end of the experiment. When a single experiment reported several responses related to the same organism (e.g., growth, calcification and metabolism of the same organism were reported simultaneously), all responses were included.
Reproducibility	The methods of data collection and analysis are presented in the Methods section in detail. All data have been deposited on Zenodo digital repository at https://doi.org/10.5281/zenodo.10198734. All code y have been deposited on Zenodo digital repository at https://doi.org/10.5281/zenodo.10198752.
Randomization	Not applicable - this is a meta-analysis based study.

Blinding	Not applicable - this is a meta-analysis based study.

Did the study involve field work? ☐ Yes ☒ No

Reporting for specific materials, systems and methods

We require information from authors about some types of materials, experimental systems and methods used in many studies. Here, indicate whether each material, system or method listed is relevant to your study. If you are not sure if a list item applies to your research, read the appropriate section before selecting a response.

Materials & experimental systems

n/a	Involved in the study
☒	☐ Antibodies
☒	☐ Eukaryotic cell lines
☒	☐ Palaeontology and archaeology
☒	☐ Animals and other organisms
☒	☐ Clinical data
☒	☐ Dual use research of concern
☒	☐ Plants

Methods

n/a	Involved in the study
☒	☐ ChIP-seq
☒	☐ Flow cytometry
☒	☐ MRI-based neuroimaging

Plants

Seed stocks	Not applicable - this is a meta-analysis based study.
Novel plant genotypes	Not applicable - this is a meta-analysis based study.
Authentication	Not applicable - this is a meta-analysis based study.
